# Left atrial appendage occlusion vs standard of care in high stroke risk atrial fibrillation patients ineligible for anticoagulation: COMPARE-LAAO

**DOI:** 10.1007/s12471-025-02005-7

**Published:** 2025-12-16

**Authors:** Errol W. Aarnink, Marina F. M. Huijboom, Frank van der Kley, Richard Folkeringa, Joris R. de Groot, Pepijn H. van der Voort, Yuri Blaauw, Marisevi Chaldoupi, Jeroen Stevenhagen, George J. Vlachojannis, Nicolas M. van Mieghem, Martin J. Swaans, Vincent F. van Dijk, Marcel G. Dijkgraaf, Ewoud J. van Dijk, Jan G. P. Tijssen, Lucas V. A. Boersma

**Affiliations:** 1 Department of Cardiology, St. Antonius Hospital, Nieuwegein, The Netherlands; 2 Department of Cardiology, Leiden University Medical Center, Leiden, The Netherlands; 3 Department of Cardiology, Medical Center Leeuwarden, Leeuwarden, The Netherlands; 4 Department of Cardiology, Amsterdam University Medical Center, location AMC, Amsterdam, The Netherlands; 5 Department of Cardiology, Catharina Hospital, Eindhoven, The Netherlands; 6 Department of Cardiology, University Medical Center Groningen, Groningen, The Netherlands; 7 Department of Cardiology, Maastricht University Medical Center, Maastricht, The Netherlands; 8 Department of Cardiology, Medical Spectrum Twente, Enschede, The Netherlands; 9 Department of Cardiology, University Medical Center Utrecht, Utrecht, The Netherlands; 10 Department of Cardiology, Erasmus University Medical Center, Rotterdam, The Netherlands; 11 Department of Epidemiology and Data Science, Location AMC, Amsterdam University Medical Center, Amsterdam, The Netherlands; 12 Department of Neurology, Radboud University Medical Center, Nijmegen, The Netherlands; 13 Department of Cardiology, Clinical Epidemiology & Biostatistics, Amsterdam University Medical Center, Amsterdam, The Netherlands; 14 Department of Neurology, University Medical Center Utrecht, Utrecht, The Netherlands

**Keywords:** Atrial fibrillation, Left atrial appendage occlusion, Ischemic stroke, Oral anticoagulation, Bleeding risk

## Abstract

**Introduction:**

The left atrial appendage is the dominant source of cardioembolic stroke in patients with atrial fibrillation (AF). Contemporary guidelines recommend considering left atrial appendage occlusion (LAAO) in AF patients contraindicated to oral anticoagulation therapy (OAC), but randomized controlled trial (RCT) data for this subpopulation are lacking.

**Methods:**

COMPARE LAAO was designed as an event-driven, multicenter, prospective, randomized, open, blinded endpoint (PROBE) trial that randomized AF patients with an increased thromboembolic risk and a contraindication to OAC 2:1 to LAAO or standard-of-care (SOC). The co-primary endpoints comprised 1) time to first occurrence of ischemic/hemorrhagic/undetermined stroke and 2) time to first occurrence of all-cause stroke/TIA/SE. The trial aimed to enroll 609 patients.

**Results:**

After randomization of 69 patients, the trial was terminated prematurely by the sponsor due to a slow inclusion rate. Results are discussed briefly without formal statistical testing. All-cause stroke occurred in 7/48 and 2/21 patients randomized to LAAO and SOC, respectively. According to the as-treated principle, all-cause stroke occurred in 5/41 and 4/28 patients treated with LAAO and SOC. The composite of all-cause stroke/TIA/SE occurred in 10/48 and 4/21 patients randomized to and 8/41 and 6/28 patients treated with LAAO and SOC.

**Conclusion:**

Insufficient statistical power of COMPARE LAAO impedes drawing any conclusions. Among other factors, the loss of perceived clinical equipoise among physicians proved problematic for successful trial completion. Conducting an RCT on LAAO vs SOC in OAC-ineligible patients appears infeasible globally, which threatens to preclude reimbursement in the Netherlands for these patients that have no proven alternative.

**Supplementary Information:**

The online version of this article (10.1007/s12471-025-02005-7) contains supplementary material, which is available to authorized users.

## Introduction

Atrial fibrillation (AF) is associated with an increased risk of ischemic stroke, and oral anticoagulation therapy (OAC) is indicated in AF patients at increased risk of stroke [1]. Left atrial appendage occlusion (LAAO) may contribute to stroke prevention in the context of AF, especially in patients with a contraindication to (long-term) OAC. The rationale for LAAO mainly originates from observational data showing thrombus in the LAA after thromboembolic events. Randomized trials (RCTs) have shown non-inferiority of LAAO for the composite of stroke, transient ischemic attack (TIA), systemic embolism (SE), and death compared to vitamin K antagonists (PROTECT-AF/PREVAIL) [2]. More recently, the PRAGUE-17 RCT achieved non-inferiority for a net clinical benefit endpoint of thromboembolic events, bleeding, and death, when comparing LAAO to direct oral anticoagulants [3]. The benefit of LAAO lies in the potential for reduced bleeding, as it may allow long-term discontinuation of OAC following the procedure. Large-scale registries have endorsed this promise, showing significant relative risk reductions in both thromboembolic and bleeding events compared to acknowledged risk-score derived expected event rates in patients who discontinued OAC after LAAO [4, 5]. Based on this evidence, contemporary guidelines provide class IIa [6] and IIb [1] recommendations for LAAO in OAC-ineligible patients, but these recommendations are based on randomized data on patients tolerating OAC. Currently, there are no published RCTs on the effectiveness and safety of LAAO in patients with strict contraindications to OAC.

## Methods

The “COMPARing Effectiveness and safety of Left Atrial Appendage Occlusion to standard of care for atrial fibrillation patients at high stroke risk and ineligible to use oral anticoagulation therapy” (COMPARE LAAO) trial was designed as an event-driven, multicenter, prospective, randomized, open, blinded endpoint (PROBE) trial. Fifteen Dutch hospitals with cardiothoracic surgery back-up committed to participating. Patients were 2:1 randomized to either LAAO or standard of care (SOC), comprising antiplatelet therapy or no antithrombotic therapy. The rationale and design have been published previously [7], and the trial was preregistered (ClinicalTrials.gov; NCT04676880). Patients were eligible for the trial if they had atrial fibrillation or flutter and a guideline indication for OAC (CHA_2_DS_2_-VASc ≥ 2 for men and ≥ 3 for women), but were deemed unsuitable for the long-term use of OAC as determined by the referring physician team. Main exclusion criteria included unsuitable LAA anatomy, contraindications to catheterization, transesophageal echocardiography, or post-procedural antiplatelet therapy, significant valve disease, and heart failure (i.e. left ventricular ejection fraction < 31% and/or NYHA 3-4). The co-primary efficacy outcomes were composed 1) time to first occurrence of ischemic or hemorrhagic or undetermined stroke and 2) time to first occurrence of the composite of all-cause stroke, TIA, and SE. All prespecified clinical outcomes were adjudicated by an independent Clinical Event Committee that was blinded for treatment allocation (see the design paper for the definitions). A Data Safety Monitoring Board monitored the safety of trial subjects.

The sample size calculation was based on superiority testing for LAAO vs SOC for the outcome of stroke from any cause, estimating an annual stroke rate of 6% in the standard-of-care arm with patients not taking OAC, adding up to 18% stroke rate over the expected three-year median follow-up (individual follow-up duration: 1–5 year, depending on inclusion date). With a target relative risk of 0.50, the occurrence of 72 primary outcomes should yield an 85% power of the trial, which corresponds to a sample size of 406 patients in the intervention group and 203 patients in the SOC group. Cox proportional hazards regression analysis was planned for comparing the co-primary endpoints, with plotting of Kaplan-Meier curves for visualization of incidence over time.

The study was sponsored by ZorgInstituut Nederland as part of the Veelbelovende Zorg program that aims to evaluate whether new treatment options offer improvements in patient care and should be part of reimbursed health care. Trial oversight was performed on an annual basis by ZonMW to ensure optimal progress of the trial in accordance with all regulations that apply to the program (2020006340).

## Results

The first patient was enrolled in COMPARE LAAO on 7th January 2021, during the peak of the COVID-19 pandemic. Already during the initial phase of the trial, it became apparent that the inclusion rate was slower than expected. Despite efforts to promote the trial among cardiologists and other specialties such as neurologists, gastroenterologists, and nephrologists to boost inclusions, the inclusion rate remained slow. In November 2023, it became clear that the intended sample size could no longer be achieved within due time. At that point, the sponsor decided to discontinue the funding, and enrollment was terminated. Results are therefore briefly reported without formal statistical testing, as the limited power of the trial in its current form could produce unsubstantiated conclusions. Upon reasonable request, data is available for an individual patient data meta-analysis.

### Baseline and procedural characteristics

**Fig. 1:**
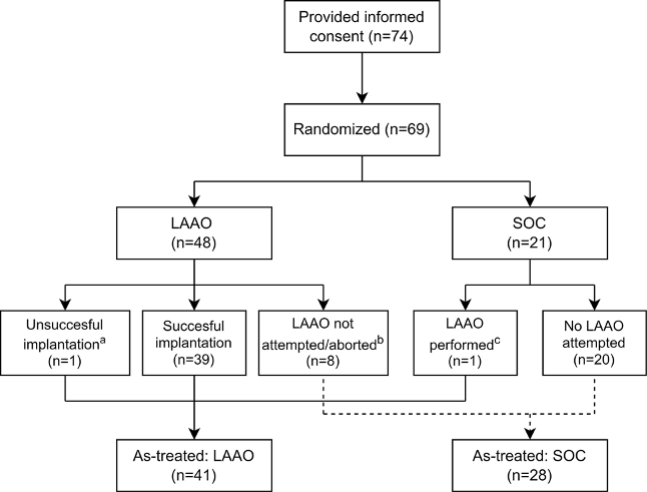
Flowchart of patient inclusion. Flowchart of study enrollment. **a** due to device dislocation; **b** due to LAA thrombus (*n* = 2), start of OAC because of stroke before LAAO (*n* = 1), withdrawal (*n* = 2) or delayed planning (*n* = 3); **c** surgical LAAO abroad. *LAAO* left atrial appendage occlusion, *SOC* standard of care

In total, 74 patients were enrolled, and 69 patients were randomized (48 to LAAO, 21 to SOC), see Fig. 1. Baseline and procedural characteristics are displayed in Tab. 1 and 2. Patients in the LAAO- and SOC-groups had a significant thromboembolic risk (CHA_2_DS_2_-VASc score: 4.1 ± 1.9 and 4.4 ± 1.6, respectively) and bleeding risk (HAS-BLED score: 3.1 ± 1.0 and 3.5 ± 0.9, respectively). A history of bleeding was the major reason for referral. Inherent to the study design, history of bleeding was highly prevalent (72% in the LAAO-group, 86% in the SOC-group), mainly consisting of intracranial hemorrhage and gastrointestinal bleeding.

**Table 1:** Main baseline characteristics

Baseline characteristics	LAAO (*n* = 48)	SOC (*n* = 21)
Age, mean (sd)	74.5 (6.2)	75.9 (5.7)
Male, *n* (%)	35 (77.8)	14 (66.7)
Body mass index, mean (sd)	25.9 (6.1)	25.5 (10.4)
History of smoking, *n* (%)	14 (31.1)	7 (33.3)
Alcohol use (> 7 units/week), *n* (%)	2 (4.5)	2 (9.5)
*Reason for referral*		
History of bleeding, *n* (%)	34 (72.3)	18 (85.7)
High risk of bleeding, *n* (%)	20 (42.6)	9 (42.9)
Recurrent bleeding, *n* (%)	11 (33.3)	11 (61.1)
*Location of previous bleeding*		
Intracranial hemorrhage, *n* (%)	24 (51.1)	9 (42.9)
Gastrointestinal bleeding, *n* (%)	7 (14.9)	6 (28.6)
Nose bleeding, *n* (%)	1 (2.1)	3 (14.3)
Intraocular bleeding, *n* (%)	1 (2.1)	0 (0.0)
Urinary tract bleeding, *n* (%)	1 (2.1)	0 (0.0)
Other, *n* (%)	2 (4.3)	1 (4.8)
*Atrial fibrillation type*		
Paroxysmal, *n* (%)	30 (66.7)	11 (52.4)
Persistent, *n* (%)	2 (4.4)	3 (14.3)
Permanent, *n* (%)	13 (28.9)	7 (33.3)
*Prior interventions*		
Ablation (PVI/CTI/other), *n* (%)	5 (11.1)	2 (9.5)
Pacemaker implantation, *n* (%)	5 (11.1)	5 (23.8)
Percutaneous coronary intervention, *n* (%)	8 (17.8)	7 (33.3)
Coronary artery bypass grafting, *n* (%)	6 (13.3)	3 (14.3)
Carotid surgery, *n* (%)	2 (4.5)	2 (9.5)
*History of thromboembolic events*		
Ischemic stroke, *n* (%)	13 (29.5)	4 (19.0)
Transient ischemic attack, *n* (%)	6 (13.6)	5 (23.8)
Systemic embolism, *n* (%)	4 (9.3)	0 (0.0)
Myocardial infarction, *n* (%)	6 (13.6)	6 (28.6)
*Comorbidities*		
Cerebral amyloid angiopathy, *n* (%)	12 (63.2)	4 (44.4)
Hereditary hemorrhagic telangiectasia,*n*n (%)	1 (5.3)	1 (11.1)
Arteriovenous malformation, *n* (%)	0 (0.0)	1 (11.1)
Congestive heart failure, *n* (%)	6 (14.3)	5 (23.8)
Hypertension, *n* (%)	38 (90.5)	17 (81.0)
Vascular disease, *n* (%)	16 (38.1)	10 (47.6)
Diabetes mellitus, *n* (%)	8 (18.6)	4 (19.0)
Renal disease, *n* (%)	3 (7.0)	2 (9.5)
Liver disease, *n* (%)	0 (0.0)	0 (0.0)
*CHA*_*2*_*DS*_*2*_*-VASc score, median [IQR]*	4 [3–5]	4 [3–5]
*HAS-BLED score, median [IQR]*	3 [3–4]	3 [3–4]
*Antithrombotic medication at baseline*		
None, *n* (%)	27 (60.0)	11 (52.4)
Aspirin monotherapy, *n* (%)	9 (20.0)	6 (28.6)
Clopidogrel monotherapy, *n* (%)	8 (17.8)	3 (14.3)
Aspirin+Clopidogrel, *n* (%)	1 (2.2)	1 (4.8)

Main baseline characteristics. PVI: pulmonary vein isolation, CTI: Cavotricuspid isthmus ablation.

**Table 2:** Main procedural characteristics

Procedural characteristics	LAAO (*n* = 48)
*Sedation*	
General anaesthesia, *n* (%)	17 (40.5)
Monitored sedation, *n* (%)	25 (59.5)
*Device type*	
Watchman FLX, *n* (%)	26 (61.9)
Size of implanted Watchman FLX, mean (sd)*	24.4 (3.7)
* No. of devices used, mean (sd)*	1.04 (0.20)
Amplatzer Amulet, *n* (%)	15 (35.7)
* Size of implanted Amplatzer Amulet, mean (sd)*	23.7 (2.6)
* No. of devices used, mean (sd)*	1.13 (0.52)
*Manufacturer closing criteria*	
PASS complete (Watchman FLX)*, *n* (%)	20 (83.3)
CLOSE complete (Amplatzer Amulet)*†, n* (%)	15 (100.0)
*Miscellaneous*	
Heparin dose, median (IQR)	7,500 (5,000–7,500)
Activated clotting time, mean (sd)	272.1 (122.7)
Duration of device implantation in minutes, mean (sd)	46.9 (19.4)
*Procedural complications*	
Pericardial effusion, *n* (%)	1 (2.4)
Cardiac tamponade, requiring pericardiocentesis, *n* (%)	1 (2.4)
Thrombus on the sheath	1 (2.4)
TEE probe complication	1 (2.4)
Device embolization, requiring percutaneous retrieval, *n*n (%)‡	1 (2.4)
TIA/stroke‡	1 (2.4)
Anesthesia complication	0 (0)
Air embolism	0 (0)
Major bleeding	0 (0)

Main procedural characteristics.

*PASS: positioning, anchoring, size of compression (10–30%), seal. In 4/24 (16.7%), the seal criterion was not fulfilled due to flow under the device (all < 5 mm).

†CLOSE: midpoint lobe distal to Cx, Lobe compressed, Orientation of the lobe in line with axis of the LAA neck, Separation between lobe and disc, Epileptical/concave disc.

‡ both complications occurred in the same patient

LAAO was successfully performed in 39/40 patients, where percutaneous LAAO was attempted (98%). The median procedure duration was 46.9 ± 19.4 min. The Watchman FLX and Amplatzer Amulet devices were implanted in 62% and 36% of cases, respectively. Most patients underwent LAAO under local anesthesia with conscious sedation to better tolerate the procedural transesophageal imaging. Single observations during the procedure included pericardial effusion, cardiac tamponade requiring pericardiocentesis, a thrombus on the sheath, and a TEE probe complication, none leading to relevant sequelae. In one case, the LAAO device migrated shortly after implantation, resulting in an ischemic stroke. The device could be percutaneously retrieved without further sequelae. No other procedural complications occurred.

### Clinical outcomes

We intended to complete at least one year of follow-up in all patients, which yielded a median follow-up duration of 379 days (IQR: 361–734 days). As several crossover situations occurred (Fig. 1) and outcomes were adjudicated by a blinded clinical event committee, outcomes are reported both according to the intention-to-treat (ITT) and as-treated (AT) principles.

**Fig. 2:**
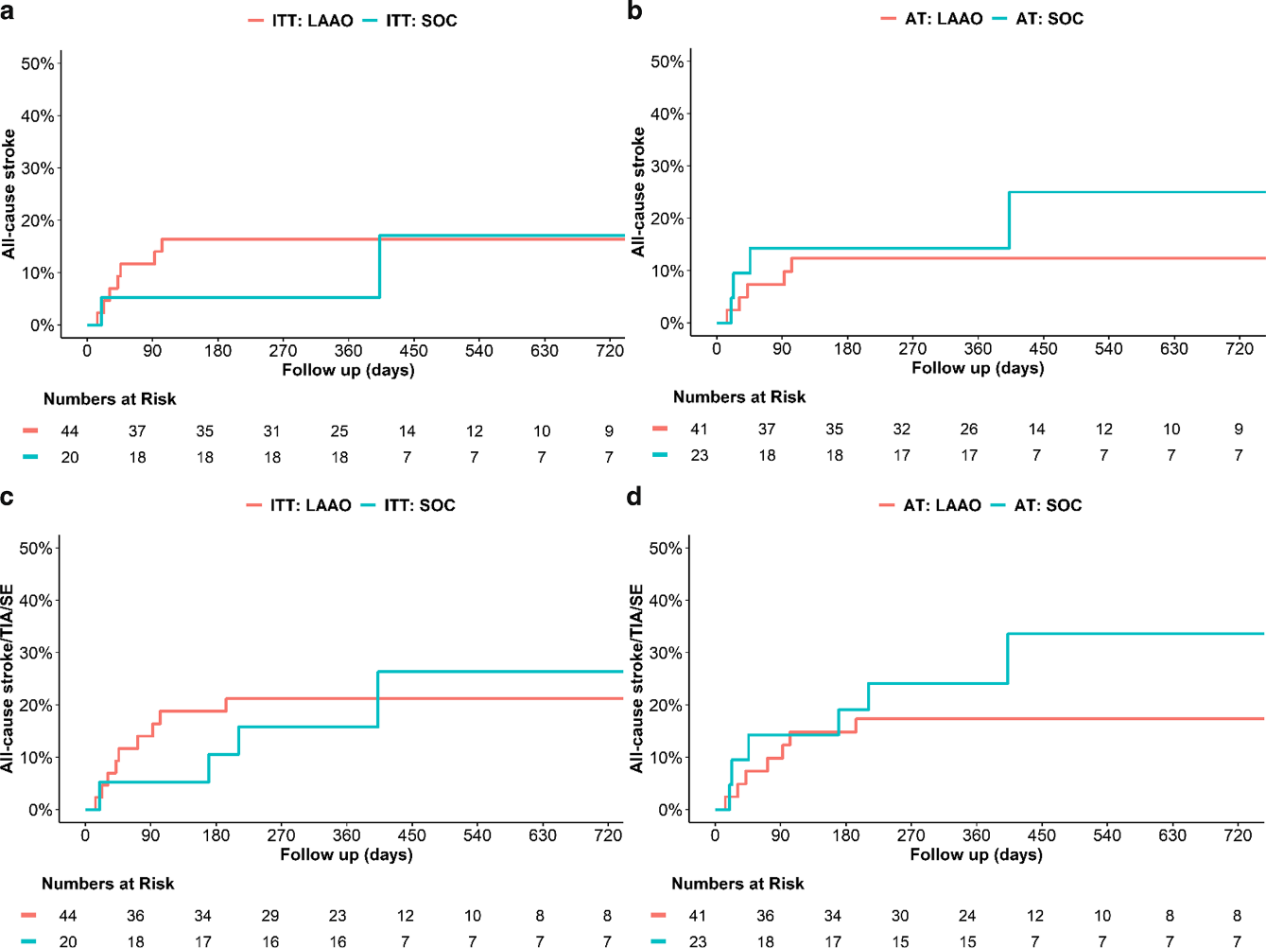
Kaplan-Meier curves for primary outcomes. Kaplan-Meier curves showing time-to-event data for all-cause stroke (Panels A and B) and the composite of all-cause stroke, TIA and SE (Panels C and D). *ITT* intention-to-treat, *AT* as-treated, *LAAO* left atrial appendage occlusion, *SOC* standard of care

In the ITT analysis, all-cause stroke occurred in 7/48 patients randomized to LAAO (15%) and 2/21 patients randomized to SOC (10%). In the AT analysis, all-cause stroke occurred in 5/41 LAAO-patients (12%) and 4/28 SOC patients (14%). The composite of all-cause stroke, TIA and SE occurred in 10/48 patients randomized to LAAO (21%) and 4/21 patients randomized to SOC (19%) with ITT analysis. In the AT analysis, 8/41 patients undergoing LAAO (20%) and 6/28 patients treated as SOC (21%) suffered from all-cause stroke, TIA, or SE. Kaplan-Meier curves for the primary outcomes are presented in Fig. 2. All primary and secondary trial outcomes are presented in Tab. 3.

**Table 3:** Adjudicated clinical outcomes per treatment arm

	Intention to treat						As treated					
	LAAO (*n* = 48)			SOC (*n* = 21)			LAAO (*n* = 41)			SOC (*n* = 28)		
	*n*	%	AER (95% CI)	*n*	%	AER (95% CI)	*n*	%	AER (95% CI)	*n*	%	AER (95% CI)
*Primary outcomes*												
All-cause stroke	7	15%	14.3% (5.8–29.5)	2	10%	7.5% (0.9–27.3)	5	12%	10.2% (3.3–23.8)	4	14%	15.2% (4.2–39.0)
Composite of all-cause stroke, TIA and SE	10	21%	22.2% (10.6–40.8)	4	19%	15.7% (4.3–40.2)	8	20%	17.7% (7.6–34.8)	6	21%	23.7% (8.7–51.7)
*Secondary outcomes*												
Ischemic stroke*	3	7%	5.7% (1.2–16.7)	1	5%	3.8% (0.1–21.1)	2	5%	3.9% (0.5–14.2)	2	8%	7.1% (0.9–25.7)
Hemorrhagic stroke	4	9%	7.5% (2.0–19.2)	1	5%	3.8% (0.1–21.0)	3	7%	5.9% (1.2–17.0)	2	8%	7.1% (0.9–25.6)
Undetermined stroke	0	0%	–	0	0%	–	0	0%	–	0	0%	–
TIA	3	7%	5.7% (1.2–16.5)	2	10%	7.9% (1.0–28.4)	3	7%	6.1% (1.3–17.7)	2	8%	6.9% (0.8–24.8)
Systemic embolism	0	0%	–	0	0%	–	0	0%	–	0	0%	–
Major bleed (BARC3-5)*†*	6	14%	11.4% (4.2–24.9)	2	10%	7.6% (0.9–27.5)	5	12%	9.9% (3.2–23.0)	3	13%	10.7% (2.2–31.4)
Minor bleed (BARC1-2)	5	11%	9.4% (3.1–22.0)	1	5%	3.8% (0.1–21.4)	5	12%	10.1% (3.3–23.6)	1	4%	3.4% (0.1–18.8)
All-cause mortality	7	16%	19.5% (9.7–34.9)	3	14%	18.5% (6.0–43.2)	7	17%	20.8% (10.4–37.3)	3	11%	16.3% (5.3–38.1)
Cardiovascular death	3	7%	–	1	5%	–	3	7%	–	1	4%	–
Non-cardiovascular death	4	9%	–	2	10%	–	4	10%	–	2	8%	–
Unspecified death	0	0%	–	0	0%	–	0	0%	–	0	0%	–
Device-related thrombus	0	0%	–	–	–	–	0	0%	–	–	–	–

Prespecified clinical outcomes per treatment arm (intention-to-treat and as-treated). All outcomes in the table were adjudicated by an independent clinical event committee that was blinded for treatment allocation. The column *n* represents number of patients with an event. AER was computed using time-to-first-event data.

*TIA* transient ischemic attack, *BARC* bleeding academic research consortium, *AER* annualized event rate.

* the ischemic stroke resulting from device dislocation was categorized as LAAO for both the ITT and AT analysis

† including hemorrhagic stroke

Follow-up imaging was performed in 37 patients at three months (95%) and 18 patients at one year (46%). No device-related thrombus (DRT) was observed. Three months after LAAO, peri-device leak (PDL) was present in 8 patients (22%). The leak was smaller than 3 mm in 7 patients (88%) and larger than 5 mm in 1 patient (13%), with all leaks persisting in the four PDL-cases where one-year imaging was performed. After one year of follow-up, OAC was started in 2 patients in the SOC arm and 2 patients in the LAAO arm. Antithrombotic strategy during follow-up is illustrated in Supplementary Fig. S1.

## Discussion

The COMPARE LAAO was designed to show superior thromboembolic event reduction of LAAO over SOC in AF patients with a high risk of stroke and a contraindication to OAC. The study failed in this intention due to a low recruitment rate and had to be terminated prematurely. A worldwide RCT with a similar design, ASAP-TOO, faced the same problem and also had to stop enrolment prematurely. For now, no statistically sound conclusions can be drawn from the COMPARE LAAO trial, as the intended sample size was not reached. However, some preliminary results should be considered. The annual ischemic stroke rate was quite well in line with the expected rate of 6% used in our sample size calculation; i.e., 3.9% and 7.1% in patients treated with LAAO or as SOC, respectively. The primary outcome of all-cause stroke occurred frequently due to the very high prevalence of hemorrhagic stroke in both treatment arms. These findings may be attributed to the severe comorbidities observed in this high-risk population. According to the present results, it is unclear if the target relative risk of 0.50 would be achieved when the sample size had been reached and if the superiority of LAAO over SOC could be demonstrated.

The slow enrolment speed of COMPARE LAAO can be attributed to various reasons. We experienced a long trial initiation phase due to introduce an intervention that was new to multiple study sites. Moreover, the COVID-19 outbreak limited capacity. Some study sites never initiated enrollment. However, the inclusion rate in initiated sites was also poor, with three factors playing a major role in our opinion.

First, strict contraindication for OAC is almost always issued by physicians from other specialties than cardiology due to increased (non-cardiac) bleeding (risk), such as hematuria, intracranial hemorrhage, or gastrointestinal bleeding history. Unfamiliarity of non-cardiac specialists with the possibility of LAAO in this population may have limited referrals. In the Netherlands, 25–34% of AF patients do not continue with DOAC use after one year, and 64% discontinue within four years of their first prescription [8, 9]. Approximately one third of discontinuations may be contributed to bleeding complications [10], corresponding to more than 50,000 patients nationally. COMPARE LAAO eligibility criteria may not always apply, but a significant proportion of these patients may have been suitable yet were not identified. Even in the American healthcare setting, where LAAO is reimbursed and performed much more often, consideration of LAAO was documented in less than a quarter of patients after a major bleeding [11].

Second, and seemingly more important, the definition of contraindications to OAC may vary between physicians, and a substantial grey area in treatment strategies exists in the AF population that has a high bleeding risk [12]. Although not recommended in international guidelines, off-label use of low-dose DOACs is often applied because of bleeding, but may come at the cost of an increased thromboembolic risk [13]. However, the choice for this strategy is understandable as physicians fear a stroke may occur if they withhold all OAC from their patient, as patients are still exposed to a significant thromboembolic risk when not taking OAC (6.7%/year, based on a median CHA_2_DS_2_-VASc score of 4 in COMPARE LAAO). Given the inclusion criterion of strict contraindication to OAC, one may also argue that absolute contraindications to OAC are rare, despite high discontinuation rates in population-based studies. Balancing between ischemic stroke or major hemorrhage may change over time—for instance after correction of some factors described in the HAS-BLED score. DOAC discontinuation may also lead to treatment with alternatives like VKA or long-term low-molecular-weight heparin. Patients with relatively low CHA_2_DS_2_-VA scores might be more eager to discontinue OAC in case of recurrent bleeding. These were not included in COMPARE-LAAO.

Last, and most important, we experienced a strong preference of both referring physicians and referred patients for the intervention group and unwillingness to be randomized to the control arm, not receiving any stroke prevention. Thus, patients often received LAAO outside of our trial, both by surgical and percutaneous approach. This potentially occurred most frequently in the patients that are most at risk for bleeding and thromboembolism, as waiting for another event in the control group may be deemed unethical. We are nonetheless unaware of the number of potential candidates that were not referred to in the first place. Furthermore, even after randomization to the control group, surgical exclusion of the LAA has occurred abroad. The similar ASAP-TOO trial (NCT02928497) also ended enrollment prematurely due to recruitment difficulties in the American healthcare setting, where nowadays approximately 200,000 LAA occluders are implanted per year [14]. Evidence for similar efficacy of LAAO to anticoagulation therapy regarding thromboembolic and mortality risk may be extrapolated to the population with a contraindication for anticoagulation, as anticoagulation therapy has already been widely proven to be superior to no anticoagulation or aspirin in atrial fibrillation patients at increased stroke risk. Moreover, with emerging (indirect) evidence for LAAO in contraindicated patients and increasingly favourable guideline recommendations for both percutaneous and surgical LAAO, consensus seems to have outpaced randomized evidence. Loss of trust in clinical equipoise among physicians is problematic for conducting a trial like COMPARE LAAO, now and in the future [15].

This impedes LAAO from becoming an acknowledged option for the prevention of thromboembolic events in the Netherlands. Currently, no reimbursement options exist, while, on the contrary, LAAO is increasingly adopted in the United States and most European countries based on the same evidence, thus leading to an inequality of healthcare delivery for Dutch patients. Since healthcare costs are expected to increase, critical appraisal of new treatment strategies is essential. On the other hand, the restrictive (monetary) policy for reimbursement in the Netherlands may hamper healthcare innovation. In the absence of upcoming RCTs in patients with a strict OAC contraindication, the position of LAAO in the Netherlands seems to have been sidelined currently, while indirect evidence for LAAO in this population seems eminent and emerging in large registries of almost 100,000 patients benefitting from this therapy [16].

Moreover, new RCTs no longer aim at contraindicated patients where reimbursement has been achieved in many countries. Instead, these studies challenge OAC use in patients without a known bleeding risk. Recently, the OPTION trial showed that in patients with AF and high stroke risk undergoing AF ablation, LAAO was non-inferior to DOAC for stroke, SE, and death, while it was superior for the safety endpoint of non-procedure-related bleeding [17]. The CHAMPION-AF (NCT04394546) and CATALYST (NCT04226547) trials with a similar endpoint strategy will show results in the near future in AF patients randomized to LAAO or DOAC. It is difficult to predict what a positive trial would do for the position of LAAO in the Netherlands, as even in the most justifiable population of AF patients with bleeding problems it is not accepted for reimbursement currently.

### Limitations

In some patients with strict contraindications for OAC, the use of DAPT may entail an unacceptable bleeding risk, such as in patients with prior intracranial hemorrhage (ICH) and Cerebral Amyloid Angiopathy (CAA). During the conduction of the trial, post-procedural single antiplatelet therapy instead of DAPT was allowed, as referring neurologists frequently advised against the use of DAPT in those patients. All five ICHs occurred in patients with a history of CAA, of whom two patients used DAPT following LAAO. The patient-specific balance between thrombotic and bleeding risk seems to mandate a tailored approach, but robust randomized data on optimal antithrombotic strategy following LAAO is lacking—especially for subgroups such as patients with prior ICH due to CAA. Upcoming randomized trials (CLEARANCE; NCT04298723, STROKECLOSE; NCT02830152, A3ICH; NCT03243175) investigate LAAO vs SOC in patients with prior ICH. Novel LAAO devices aim for improved endothelialization and lower DRT risk, which may abate the need for post-procedural antithrombotic medication.

PDL was a relatively frequent finding in the COMPARE LAAO study, observed in 22% of patients at three-month follow-up. This aligns with previous reports, where PDL has been documented in 11–57% of cases, depending on definition and imaging modality. An elevated risk of thromboembolic events, particularly when PDL exceeds 5 mm, has been reported [18]. In our cohort, one patient with PDL experienced a TIA, while no other thromboembolic events occurred among patients with PDL.

Furthermore, because we specifically enrolled patients with high bleeding risk who had strict OAC contraindications, often accompanied by serious comorbidities, the mortality rate in our population was high (10/69 patients), similar to that observed in the EWOLUTION trial. OAC-ineligible patients typically present with both high bleeding risk and severe comorbidities, which may compromise survival. As early post-procedural mortality could lead to futility, adequate mortality risk assessment should be performed before considering LAAO, for instance, by applying comorbidity-based mortality risk assessment [19].

## Conclusion

COMPARE-LAAO did not achieve its intended purpose due to a slow inclusion rate. This may in part be attributed to loss of perceived clinical equipoise for LAAO and SOC in patients deemed contraindicated to OAC. Currently, it is open for discussion whether, despite the presence of high-quality observational data, the absence of evidence from randomized studies should prevent the therapy of left atrial appendage closure in the Netherlands for patients with a contraindication to OAC. We believe that LAAO should be offered and reimbursed in patients who have no safe and effective alternative for stroke prevention. This may be feasible under strict patient selection criteria, performed in a small number of dedicated implanting centers, in line with good clinical practice in most other European countries, to avoid regional disparity and inequality in treatment options for this vulnerable patient population. Randomized controlled outcomes in such patients with a strong contraindication to OAC are unlikely to be obtained anymore, as the field has already embraced LAAO for this situation. New RCT evidence investigating the non-inferiority of LAAO to DOAC for thromboembolism and superiority for bleeding is imminent and may reopen the discussion on the future of LAAO in the Netherlands.

### What’s new

- Guidelines provide favourable recommendations for LAAO in OAC-ineligible patients, but there are no published RCTs on the effectiveness and safety of LAAO in patients with strict contraindications to OAC
- In COMPARE LAAO, AF patients with an increased thromboembolic risk and a contraindication to OAC were randomized 2:1 to LAAO or standard-of-care
- Slow inclusion rate led to premature termination of the trial by the sponsor, which impedes drawing statistical conclusions
- Loss of perceived clinical equipoise between LAAO and SOC in this population questions the feasibility of a future RCT, as consensus seems to have outpaced randomized evidence

## Supplementary Information

Supplementary appendix
