# Supplementary material for: Left atrial appendage occlusion vs standard of care in high stroke risk atrial fibrillation patients ineligible for anticoagulation: COMPARE-LAAO
Source: Neth Heart J. 2025 Dec 16;34(1):18–26. doi: 10.1007/s12471-025-02005-7 (PMC12779848; doi:10.1007/s12471-025-02005-7)
Supplement: Supplementary file 1 — Supplementary appendix [file 12471_2025_2005_MOESM1_ESM.docx]

**SUPPLEMENTARY APPENDIX**

**Figure 1:** Antithrombotic strategy during follow-up

**
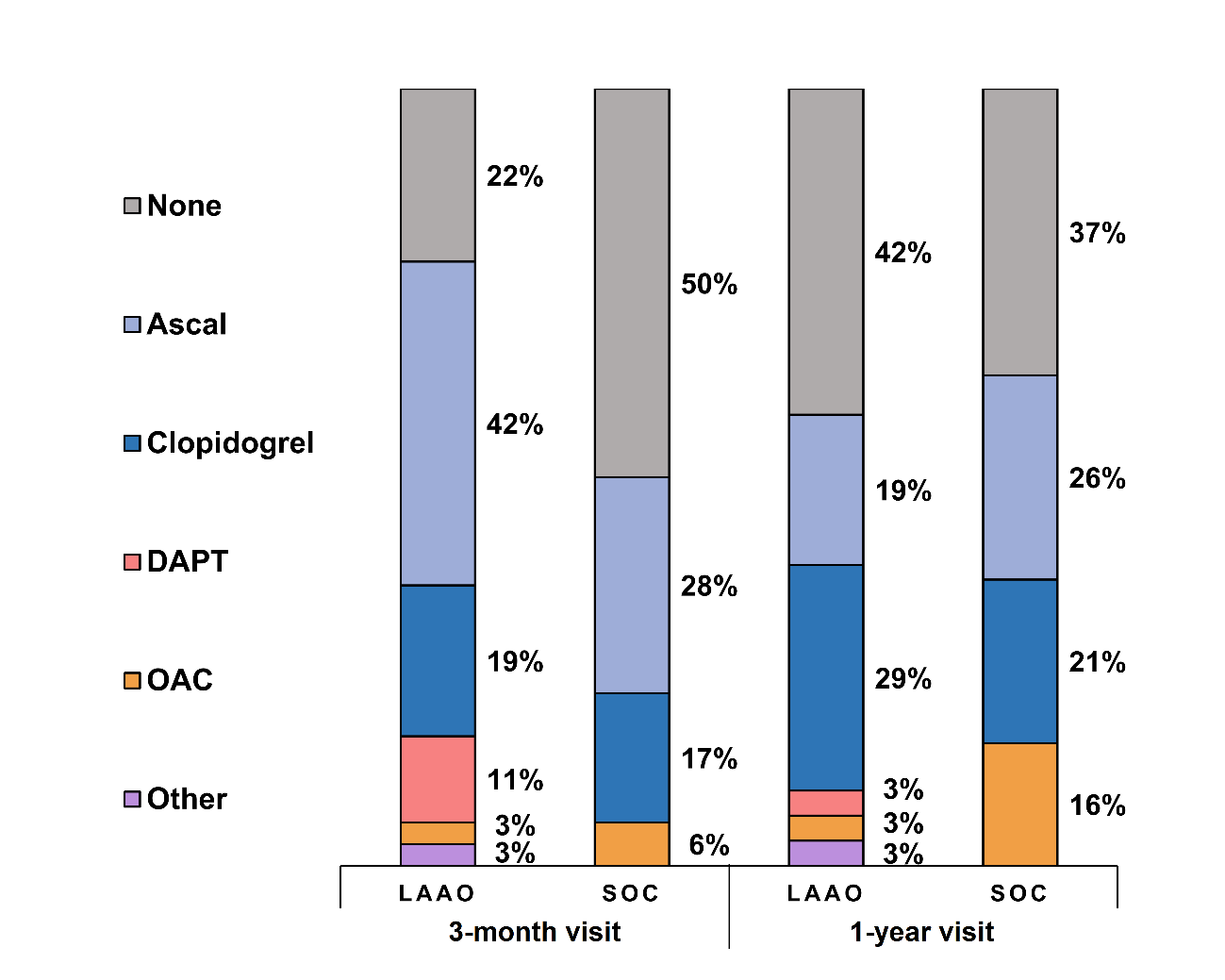
**

*Antithrombotic medication use according to as-treated analysis, per time point. Due to limited data, visits after the 1-year visit are not displayed. DAPT: dual antiplatelet therapy, OAC: oral anticoagulation*
